# Insights from GWAS, TWAS, and DEG analysis: CDKN2B-AS1, MAP3K4, and P2RX2-encoded P2X2 receptor identified as potential therapeutic targets for ischemic heart disease in Russian adults and long-living individuals

**DOI:** 10.3389/fcvm.2026.1839477

**Published:** 2026-08-13

**Authors:** Veronika Daniel, Natalia Romanova, Aleksandra Mamchur, Anna Nartova, Gerel Abushinova, Mikhail Ivanov, Marat Ezhov, Alexey Meshkov, Natalia Gomyranova, Vladislav Vasiliev, Ruslan Latypov, Yuliya Vorobieva, Olga Gostischeva, Ulyana Chubykina, Timur Gurtsiev, Yuliya Aksenova, Madina Komarova, Nayana Kumar, Ekaterina Maralova, Andrey Shingaliev, Sergey Mitrofanov, Vladimir Yudin, Anton Keskinov, Olga Tkacheva, Veronika Skvortsova, Sergey Boytsov, Sergey Yudin, Daria Kashtanova

**Affiliations:** 1 Federal State Budgetary Institution «Centre for Strategic Planning and Management of Biomedical Health Risks» of the Federal Medical and Biological Agency (Centre for Strategic Planning, of the Federal Medical and Biological Agency), Moscow, Russia; 2 Federal State Budgetary Institution «National Medical Research Center for Cardiology Named After Academician Yevgeniy Chazov» of the Ministry of Health of the Russian Federation, Moscow, Russia; 3 Federal State Budgetary Institution «National Medical Research Center for Therapy and Preventive Medicine» of the Ministry of Healthсare of the Russian Federation, Moscow, Russia; 4 Russian Clinical Research Center for Gerontology of Pirogov Russian National Research Medical University of the Ministry of Healthcare of the Russian Federation, Moscow, Russia; 5 Federal Medical Biological Agency (FMBA of Russia), Moscow, Russia

**Keywords:** DEG (differentially expressed gene) analysis, GWAS—genome-wide association study, ischemic heart disease (IHD), polygenic risk score (PRS), TWAS—transcriptome-wide association study

## Abstract

**Background:**

The purpose of this study was to investigate genetic risk factors associated with ischemic heart disease (IHD) in Russian adults, to develop prognostic PRS models for IHD risk stratification, and to gain a better understanding of molecular mechanisms underlying IHD through a transcriptome analysis.

**Materials and methods:**

The study analyzed data from three independent cohorts: a population sample of 69,500 individuals, a cardiac sample of 5,875 IHD patients, and a sample of 2,870 long-living adults. To identify genomic loci associated with IHD, a GWAS was performed, and its findings were used to develop a PRS model for the IHD phenotype. Additionally, a transcriptome analysis was conducted.

**Results:**

The GWAS for IHD adjusted for sex, age, and the first ten principal component accounting for population structure identified 75 variants, with 67 located at 9p21.3. Based on the GWAS findings, PRS models were developed and validated for IHD. The risk of IHD adjusted for sex, age, and BMI was the lowest in the sample of long-living adults. The TWAS revealed a significant association between IHD and increased CDKN2B expression in the aorta. According to the DEG results, the CDKN1A gene, a member of the same gene family as CDKN2B, was significantly hypoexpressed in the lower limb veins of patients with IHD.

**Conclusion:**

PRS models offer great benefits for IHD risk prediction, particularly in individuals at the e tremes of the heritable risk distribution. lncRNAs, such as CDKN2BAS1 and lncRNA-MAP3K4, as well as P2X2 receptors, could be potential therapeutic targets for IHD.

## Graphical Abstract

**Figure:**
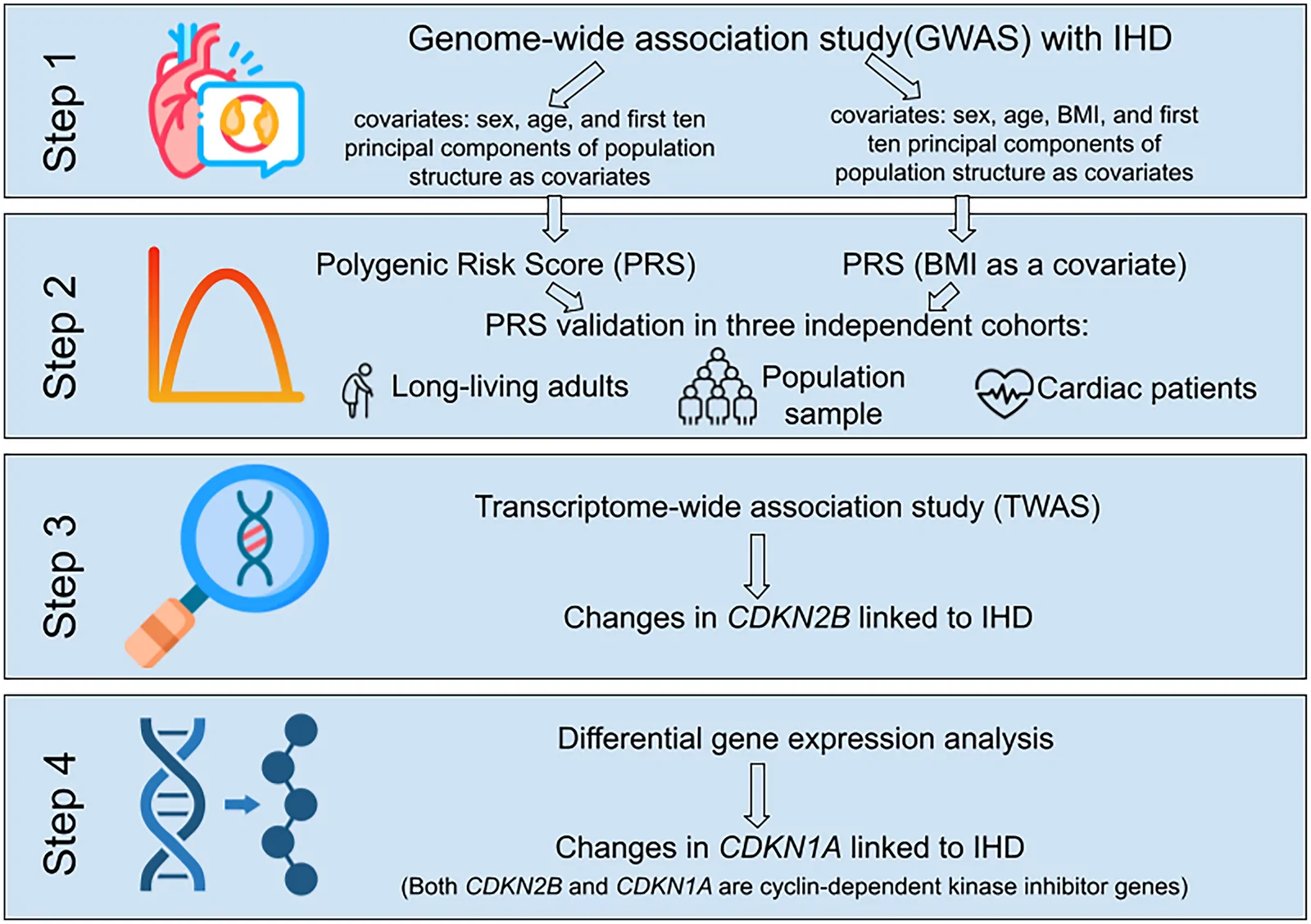

## Key findings

1. 5 variants previously not associated with IHD were identified: rs141766382 in an intron of the *MAP3K4* gene, rs7314492 in the 5′UTR of the *P2RX2* gene, chr12:110905890:A>AATAT in an intron of the *CCDC63* gene, chr4:82798086 CG>C in an intron of the *SCD5* gene, and rs56069116 in an intron of the *TBKBP1* gene*.* The *CDKN2B-AS1*, *LPA*, *MAP3K4*, *NR5A1*, *CCDC63*, *SCD5*, *TBKBP1*, *AGPAT4*, and *P2RX2* genes were identified as the most significant in the GWAS.
2. Variants in the *CDKN2B-AS1* gene were associated with a small but statistically significant decrease in HDL levels (rs1333040, rs7857345) and an increase in LDL levels (rs1333047), as well as more severe damage to the coronary vascular bed (rs1333040, rs1333047).
3. Long non-coding RNAs (CDKN2B-AS1 and lncRNA-MAP3K4) and P2X2 receptors were identified as promising therapeutic targets for IHD.
4. Two PRS models for IHD (with and without BMI adjustment) were developed and validated.
5. TWAS identified a significant association between IHD and increased *CDKN2B* gene expression in the aorta, which was not confirmed by differential expression analysis.
6. The differential expression analysis showed that the *CDKN1A* gene, from the same family as the *CDKN2B* gene, was significantly hypoexpressed in lower limb veins of IHD patients.

## 1 Introduction

Ischemic heart disease (IHD) occurs when the heart receives an insufficient blood supply. High prevalence of IHD and the related disability and mortality represent significant global medical and societal challenges, which necessitate improved diagnostic, therapeutic, and preventive strategies (1, 2).

The development of IHD involves complex pathological genetic mechanisms influenced by environmental factors, lifestyle, and genetic predisposition. Numerous risk assessment tools are available for evaluating IHD risk. Currently, risk assessment primarily relies on clinical risk factors analyzed through models such as SCORE2 (3), RF-CL, RF-CL (4), and CACS-CL (4). However, these evaluations are based on risk factors that typically manifest after the disease has developed. It could be highly advantageous to undertake an early risk assessment for IHD at a time when risk factors have not yet contributed to the onset of the disease.

The advent of new-generation sequencing has facilitated affordable genetic testing, while the accumulation of extensive data has helped identify genetic markers of IHD and the associated risk factrors, including dyslipidemia, diabetes mellitus, arterial hypertension, inflammation, and oxidative stress. In 2002, Ozaki et al. identified variants in the lymphotoxin-alpha (*LTA*) gene that contributed to the genetic predisposition to myocardial infarction (5). In 2007, the first genome-wide association study (GWAS) was published in the Nature journal, which provided breakthrough insights into the genetic basis of IHD. The study identified a number of SNPs associated with an increased risk of IHD, including variants not linked to traditional risk factors. The 9p21.3 locus was reported as a key genetic marker of IHD (6). Polygenic risk score (PRS) models take into account complex gene interactions and their combined effect on the phenotype and thereby facilitate the assessment of genetic predisposition to polygenic diseases, including IHD. The introduction of PRS models into clinical practice expands the opportunities for personalized medicine.

Large-scale studies have demonstrated the benefits offered by genomic screening for IHD risk stratification across different age groups (7). Studies incorporating genomic risk scores derived from multiple GWAS findings, i.e., meta-analysis (metaGRS), have shown that individuals in the highest quintile of metaGRS are at a markedly higher risk of developing IHD, with a hazard ratio of 4.17 (95% CI: 3.97–4.38) compared to those in the lowest quintile (8). It is important to recognize that the predictive performance of PRS models varies across different age groups. Studies have shown that PRS models for myocardial infarction (MI) demonstrate the highest predictive performance in patients under 50 years of age, whereas traditional risk factors are more predictive in older populations (9). Interestingly, polygenic traits affect the penetrance of monogenic cardiovascular disorders. For example, among carriers of pathogenic mutations in *LDLR*, *APOB*, and *PCSK9*, the odds ratio (OR) for IHD ranged from 1.3 (95% CI: 0.39–4.32) in the low PRS group to 12.61 (95% CI: 2.96–53.62) in the high PRS group, compared to individuals without monogenic mutations and with medium PRS (10).

Current advanced technologies enable the assessment of both genetic predisposition to IHD and the related risk factors at the genomic level through a methodology known as multi-PRS. The key advantage of multi-PRS lies in its ability to analyze multiple independent genetic pathways contributing to IHD through various mechanisms and their interactions. By determining the risk structure, multi-PRS supports the development of personalized prevention strategies for IHD. A 2023 study demonstrated that artificial intelligence models incorporating multiple polygenic risk scores outperform traditional PRS models in predicting the risk of cardiovascular disease (11). Patel et al. have demonstrated the potential of multi-PRS as a valuable tool for cardiac genetics (12).

The 2025 European Society of Cardiology (ESC) consensus statement, published in the European Heart Journal, highlights the benefits of modern PRSs for expanding the applicability of genetic risk assessment to younger patients. The ESC also recognizes the opportunities offered by integrating PRSs with metabolomic data and imaging findings. However, wider clinical implementation of PRSs necessitates addressing population biases, conducting rigorous randomized controlled trials with “hard” clinical endpoints, and resolving various ethical and legal challenges (13).

GWAS helps identify disease-associated single nucleotide polymorphisms (SNPs). However, to gain a deeper understanding of the molecular mechanisms underlying the disease, it is essential to determine how these SNPs influence gene transcription. Transcriptome analysis, such as transcriptome-wide association studies (TWASs), addresses this need by identifying the connection between genetic variants and changes in gene expression levels (14, 15). Numerous TWASs for IHD have been published recently due to the utility of the resulting data in the identification of novel biomarkers and potential therapeutic targets for the disease (16). Integrating a TWAS with differential gene expression (DEG) analysis enables detecting genes, which exhibit significant changes in expression across the examined groups, and correlating these changes to SNPs, which exert regulatory effects through expression quantitative trait loci (eQTL). Zhao et al. identified the *FGD6* and *PPAP2B* genes as candidate genes contributing to a high risk of atherosclerosis. The expression of these genes is influenced by cis-eQTLs in coronary arteries, which underlies their association with IHD (14).

This study presents findings from a GWAS for IHD and PRS models developed based on these findings. PRS models facilitate effective individual risk assessment and timely targeted prevention of IHD, which improves patients’ quality of life and reduces mortality from IHD at the population level. The transcriptome analysis provided a deeper understanding of the molecular mechanisms underlying the development of IHD.

## 2 Materials and methods

### 2.1 Study design and participants

IHD was defined as a documented clinical diagnosis (ICD-10 codes I20–I25) and/or a history of myocardial infarction, coronary artery bypass grafting, or percutaneous coronary intervention with stenting.

Participants for this study were recruited from three independent cohorts (Table 1): a population sample, a sample of cardiac patients, and a sample of long-living adults. All participants provided written informed consent prior to their involvement in the study.

**Table 1:** Clinical characteristics of the GWAS cohorts.

Characteristic	Case (cardiac patients, *n* = 2,879)	Control (population sample, *n* = 23,634)
Age, years	68 (61–74)	50 (41–57)
Male sex, *n* (%)	1,836 (63.77%)	10,098 (42.73%)
BMI, kg/m²	28.73 (25.74–31.89)	27.02 (24.01–30.46)
BMI >30 kg/m², *n* (%)	311 (10.80%)	1,927 (8.15%)
Hypertension, *n* (%)	2,215 (76.94%)	10,286 (43.52%)
CHD, *n* (%)	2,378 (82.60%)	0 (0%)
MI, *n* (%)	1,255 (43.59%)	0 (0%)
Type 2 DM, *n* (%)	610 (21.19%)	896 (3.79%)
Stroke/TIA, *n* (%)	337 (11.71%)	237 (1.00%)
Total cholesterol, mmol/L	3.91 (3.31–4.71)	5.23 (4.60–5.93)
LDL-C, mmol/L	2.04 (1.57–2.75)	3.42 (2.78–4.07)
HDL-C, mmol/L	1.12 (0.95–1.31)	1.40 (1.19–1.67)
Triglycerides, mmol/L	1.35 (1.02–1.85)	1.21 (0.87–1.77)
Smoking, *n* (%)	1,284 (44.60%)	8,655 (36.62%)

### 2.2 Recruitment of participants for the population sample

The population sample of 69,500 (23,634 controls for GWAS and 45, 866 individuals for PRS testing) participants included individuals from various regions of Russia, recruited during an epidemiological study conducted by the Federal State Budgetary Institution *Centre for Strategic Planning and Management of Biomedical Health Risks* (CSP) of the Federal Medical and Biological Agency of Russia between 2019 and 2022. The recruitment process ensured appropriate representation across sex and age groups. The study protocols were approved by the Ethics Committee of the CSP (excerpt from protocol No. 5 dated December 28, 2020; excerpt from protocol No. 2 dated June 1, 2021) and by the Ethics Committee of the Federal State Budgetary Scientific Institution *Izmerov Research Institute of Occupational Health* (excerpt from protocol No. 6 dated July 15, 2020).

### 2.3 Recruitment participants for the sample of cardiac patients

The sample of cardiac patients comprised patients of the National Medical Research Center for Cardiology named after academician Yevgeniy Chazov, who were enrolled on an all-comers basis between 2021 and 2023 as part of the study entitled “Genetics of Cardiovascular Disease.” The sample consisted of 5,875 patients. A total of 2,879 cardiac patients with confirmed IHD were designated as cases for the GWAS, while the remaining 2,996 were used for PRS testing. The study protocol was approved by the Local Ethics Committee (protocol No. 271, dated September 27, 2021) of the Federal State Budgetary Institution National Medical Research Center for Cardiology named after academician Yevgeniy Chazov. The study is registered on ClinicalTrials.gov under the identifier NCT06253481.

### 2.4 Recruitment of participants for the sample of long-living adults

The sample of long-living adults included 2,870 patients aged 90 years and older recruited between 2019 and 2023. The study was approved by the Local Ethics Committee of the Separate structural unit Russian Gerontology Research and Clinical Centre (RGRCC) of the Federal State Autonomous Educational Institution of Higher Education Pirogov (protocol No76 dated December 9, 2023).

### 2.5 DNA extraction and tissue sampling

DNA was extracted from whole blood samples using the QIAamp DNA Mini Kit (*Qiagen*, Germany). DNA libraries for whole-genome sequencing were prepared using the Nextera DNA Flex (*Illumina*, USA) in accordance with the manufacturer's instructions. DNA sequencing and bioinformatic analysis were carried out according to the previously described protocol (17).

### 2.6 Genome-wide association study (GWAS)

The GWAS excluded variants that did not conform to Hardy-Weinberg equilibrium (*p* ≤ 1 × 10^−6^), variants with a frequency below 0.98, multiallelic variants, and variants with a minor allele frequency of 0.01. Population structure was accounted for by adjusting for the first ten principal components from principal component analysis (PCA). To identify statistically significant associations between genetic loci and IHD, logistic regression analysis was carried out within an additive genetic model, which included principal components (PC) 1–10, age, and sex as covariates for the first GWAS variation and principal components 1–10, age, sex, and BMI for the second GWAS variation, using the following equation:

$$\log\;\left(\frac{\;p}{1-p}\right)=\beta_{0}+\beta_{c}\times C+\beta_{g}\times G$$

where *β*₀ is the intercept; *β_с_* is the covariate effect vector; *β_g_* is the genotype effect vector; *C* is the covariate vector; and *G* is the genotype vector.

All calculations were performed using Statsmodels v0.12.2 (Python) and Spark Cluster parallelization. Variants with a *p*-value <5.0 × 10^−8^ (the Bonferroni-corrected genome-wide significance threshold) were considered significant for the target variable (IHD). The results were visualized as Manhattan plots using a Javascript library.

### 2.7 Polygenic risk score models (PRS)

Polymorphisms identified in the GWAS for IHD reaching a significance threshold of *p* < 5 × 10^−6^ were used for PRS conctruction accroding to the following equation:

$$\log\;\left(\frac{\;p}{1-p}\right)=\beta_{0}+\beta_{c}\times C+\beta_{g}\times G$$

where *β*₀ is the intercept; *β_с_* is the covariate effect vector; *β_g_* is the genotype effect vector; *C* is the covariate vector; and *G* is the genotype vector.

To account for population structure, the first PRS model included sex, age, and the first ten principal components as covariates, while the second PRS model variation also included BMI as an additional covariate. The models were built in iterations, with variants added sequentially until overfitting was detected, as indicated by a decline in accuracy on the test set. Ridge regression was used to calculate the model's coefficients. The dataset was randomly divided into training (80%) and testing (20%) subsets. In each iteration, the model's performance was assessed using 10-fold cross-validation on the validation subset (20% of the training subset), with the ROC AUC used as the performance metric.

### 2.8 PRS calculations

The prognostic performance of the PRS model for IHD was validated on an independent cohort comprising individuals from the population sample (participants of the epidemiological study of 2019–2022) (*n* = 24,681), IHD patients of the National Medical Research Center for Cardiology named after academician Yevgeniy Chazov recruited in 2021–2023 (*n* = 5,835), and long-living adults (*n* = 2,870) recruited by the Separate structural unit Russian Gerontology Research and Clinical Centre (RGRCC) of the Federal State Autonomous Educational Institution of Higher Education Pirogov between 2019 and 2023. Individuals whose data were used in constructing the PRS model for IHD were excluded from the validation cohort. PRS scores were calculated for each participant. Logarithm of the odds ratio for genetic risk (logOrGenetics) was used for comparison purposes. The logOrGenetics was calculated using the following function:

$$\mathrm{logOrGenetics}=\beta_{g}\times G$$

where *β_g_* is the genotype effect vector, and *G* is the genotype vector.

### 2.9 Transcriptome-wide association study (TWAS)

The results from the non-BMI-adjusted GWAS for IHD (SNPs, logistic regression coefficients, and *p*-values) and eQTL data from GTEx (18) were used as inputs for TWAS. PrediXcan (https://github.com/hakyimlab/MetaXcan; accessed July 22, 2025) was used to predict gene expression changes, expressed as *z*-scores, in seven tissue types: vascular tissue (Artery_Aorta, Artery_Coronary, Artery_Tibial), heart tissue (Heart_Atrial_Appendage, Heart_Left_Ventricle), and adipose tissue (Adipose_Subcutaneous, Adipose_Visceral_Omentum). *Z*-scores and *p*-values were calculated and corrected using the Benjamini–Hochberg procedure for multiple testing to measure the statistical significance of the predicted expression changes in each tissue type.

### 2.10 Total RNA extraction from fresh frozen tissue samples

For transcriptomic analysis, four tissue types were sampled during interventions, such as coronary artery bypass grafting and valvular and aortic surgeries: lower extremity veins (*n* = 128, including 124 with IHD), the ascending aorta (*n* = 90, including 80 with IHD), epicardial adipose tissue (*n* = 73, including 40 with IHD), and subcutaneous adipose tissue (*n* = 22, including 18 with IHD). Controls comprised patients with non-IHD cardiovascular conditions recruited at the National Medical Research Center for Cardiology named after academician Yevgeniy Chazov (protocol described above). Tissue specimens (≥3 mm × 3 mm × 3 mm, ≥0.7 g) were transferred to individual cryovials within 15 min of excision, treated with RNA Safer Reagent (Magen, China) at a ratio of 1 mL buffer per 0.1 g tissue, and inverted 2–3 times to ensure complete submersion. Following incubation at +4 °C for at least 16 h, the supernatant was removed and samples were stored at −80 °C. Total RNA was extracted from fresh-frozen tissue samples manually following a specific protocols for each tissue type: the Rneasy lipid tissue mini kit (Qiagen, Germany) was epicardial fat, the Rneasy fibrous tissue mini kit (Qiagen, Germany) was used for the ascending aorta and veins of the lower limbs, and the Rneasy universal mini kit (Qiagen, Germany) was used for subcutaneous fat, in accordance with the manufacturer's instructions.

Bioprep-24 (Hangzhou Allsheng Instruments Co., Ltd.) was used for tissue homogenization. DNase I from the RNase-Free DNase Set (Qiagen, Germany) was used for DNA removal. RNA was eluted in 50 μL of RNase-free water. RNA was quantified using the Qubit RNA HS Assay Kit (Thermo Fisher Scientific Inc., USA) and the Qubit 4.0 fluorometer (Thermo Fisher Scientific Inc., USA), with purity assessed using the NanoDrop 8000 (Thermo Fisher Scientific, USA). The RNA integrity number (RIN) was determined using the 4200 TapeStation, an automated electrophoresis system (Agilent Technologies, USA), and the RNA ScreenTape Assay reagent kit (Agilent Technologies, USA).

### 2.11 Transcriptome library preparation and sequencing

The Illumina TruSeq Stranded Total RNA reagent kit was used for transcriptome library preparation, and TruSeq RNA UD Indexes were used to avoid cross contamination. Two replicates of each sample were created to serve as technical Infinite F Nano Plus (Tecan, Switzerland) was used for library quantification. The Agilent HS D1000 ScreenTape Reagents (Agilent Technologies, USA) and the 4200 TapeStation (Agilent Technologies, USA) were used for library sizing. The Freedom EVO platform with robotic functionality (Tecan, Switzerland) was used for pooling. The Agilent HS D1000 ScreenTape Reagents and the 4200 TapeStation (Agilent Technologies, USA) were used for polling quality assessment. The NovaSeq 6000 was used for whole-genome sequencing, utilizing the S2 Reagent Kit (200 cycles) (Illumina, USA) for 2 × 100 bp paired-end reads per replicate.

### 2.12 Differential gene expression analysis

Differential gene and dsRNA expression between IHD patients and controls was analyzed in R using the DESeq2 software package (19). Differential gene expression was considered significant at *p*_adj <0.05, i.e., the *p*-value of differences adjusted for multiple testing using the Benjamini-Hochberg procedure, and |log2FC|≥2.

Over-representation analysis (ORA) was performed for differentially expressed genes using the KEGG_2021_Human database and the list of genes, which were found significant after multiple testing correction.

### 2.13 Statistical analysis

Statistical analysis was performed using Python v3.12.7. Descriptive statistics were presented as medians and interquartile ranges for quantitative variables and as absolute frequencies and proportions for qualitative variables. Intergroup comparisons were performed using the Mann–Whitney *U* test for continuous variables and Pearson's chi-squared test for categorical variables. Multiple comparisons were adjusted using the Benjamini–Hochberg procedure. The null hypothesis was rejected at *p* < 0.05.

## 3 Results

### 3.1 Genome-wide association study

To study the genetic architecture of IHD, genome-wide and transcriptome-wide association studies were conducted in three independent cohorts, and the findings were used in the construction and validation of PRS models.

The GWAS for IHD integrated genetic data from the population sample and cardiac patients using two distinct covariate adjustments: the first encompassing sex, age, and the first ten principal components, and the second additionally adjusting for BMI. For both adjustments, the GWAS cohorts comprised 2,879 cases (cardiac patients with confirmed IHD) and 23,634 population controls (individuals without IHD). Clinical characteristics of the cohorts are provided in Table 1. The population sample in GWAS included participants aged 18 to 84 years (median age = 50 years; men = 42.7%). The sample of cardiac patients in GWAS included patients aged 22–97 years (median age = 68 years; men = 63.8%).

**Figure 1:**
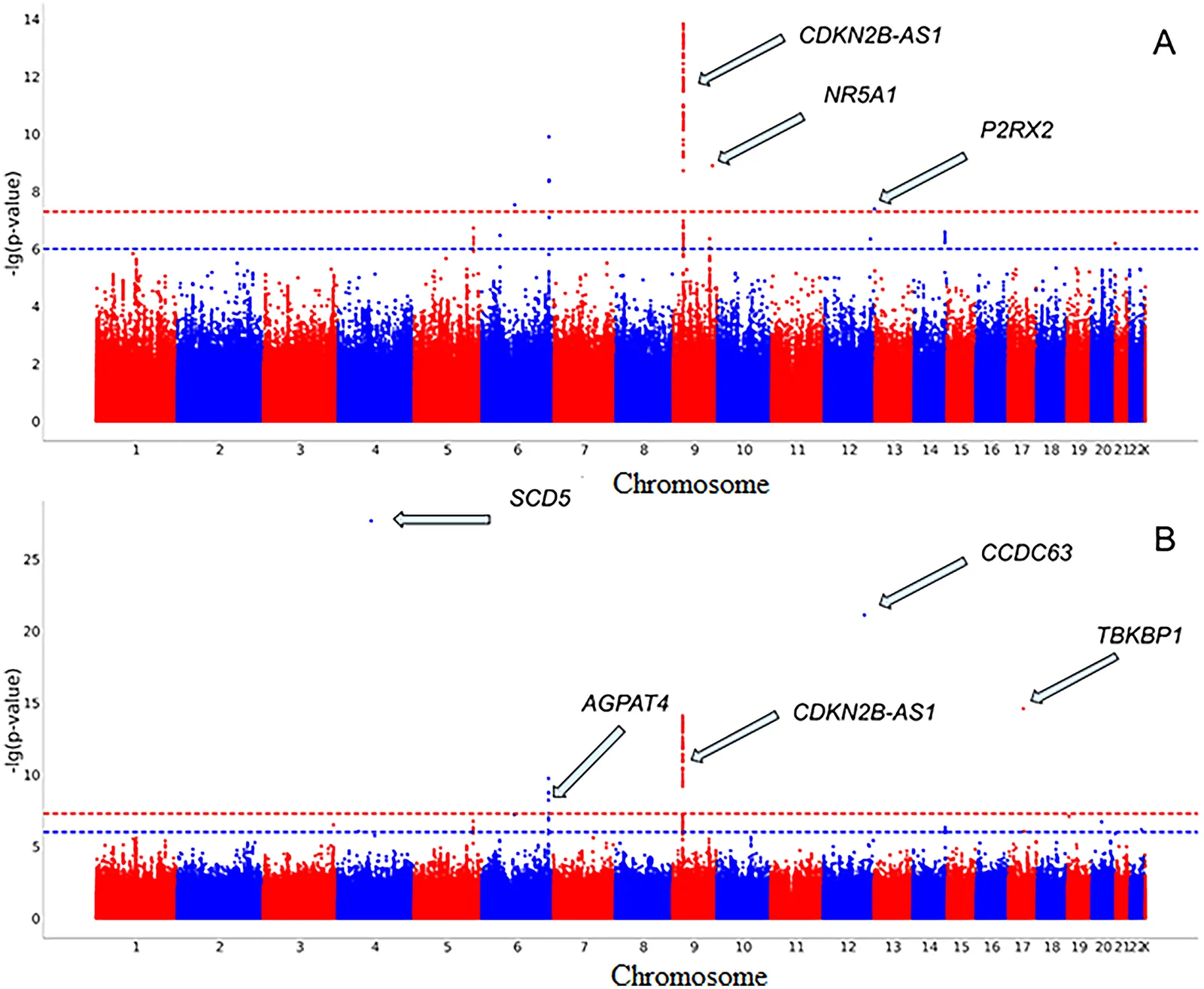
GWAS results. **(A)** GWAS results for IHD with sex, age and the first ten principal components as covariates. (B) GWAS results for IHD with sex, age, the first ten principal components, and BMI as covariates. Manhattan plots showing −log10(*p*-values) for the detected variants. The red dashed line shows the Bonferroni threshold [−log10(5 × 10^−8^)]. The blue dashed line shows the −log10(5 × 10^−6^) threshold. The arrows denote genes in which genome-wide significant variants were detected.

The first GWAS variation identified 75 polymorphisms with genome-wide significance, 67 of which were localized in a single cluster on chromosome 9 (Figure 1A; Supplementary Table S1 and Figure S1). Only five of the identified variants were deletions or insertions capable of causing frame shifts. These variants may have a significant effect on protein structure and function. It should be noted that short-read sequencing technologies have a limited accuracy for the detection of indel variants.

The addition of BMI as a variant in the second GWAS did not have a significant effect on the findings: 77 polymorphisms were identified, with 68 also located at 9p21.3 (Figure 1B; Supplementary Table S2 and Figure S2). However, variants in *NR5A1* (rs113733516, 9q33.3) and *P2RX2* (rs7314492, 12q24.33) were no longer significant in the BMI-adjusted GWAS, while other variants were unique and specific to this GWAS variation, such as rs55754459 in the 3′UTR of the *AGPAT4* gene (at 6q26), chr12:110905890:A>AATAT in an intron of the *CCDC63* gene (at 12q24.11), chr4:82798086:CG>C in an intron of the *SCD5* gene (at 4q21.22), and rs56069116 in an intronic region of the *TBKBP1* gene (at 17q21.32).

#### 3.1.1 Effect of CDKN2B-AS1 gene variants on lipid metabolism and IHD severity

Cluster analysis of the correlation matrix for *CDKN2B-AS1* gene variants (Supplementary Table S1) with genome-wide significance three variants—chr9_22083405 C>T (rs1333040), chr9_22087474T>C (rs7857345), and chr9_22124505 A>T (rs1333047), which are inherited independent from one another (Supplementary Figure S3). These variants had no effect on cholesterol or triglyceride levels. Homozygous carriage of the alternative alleles for chr9_22083405 C>T or chr9_22087474T>C exhibited a small but statistically significant decrease in high-density lipoprotein (HDL) levels (*p* = 0.001). Carriage of one or two copies of the A>T variant at chr9_22124505 was associated with reduced HDL levels (*p* < 0.001). Carriers of an alternative T>C allele at chr9_22087474 showed a slight increase in low-density lipoprotein (LDL) levels (*p* = 0.04).

In addition, the presence of one or two alternative C>T alleles at chr9_22083405 or A>T at chr9_22124505 was associated with a history of coronary artery bypass grafting and, therefore, more severe damage to the coronary vascular bed (*p* < 0.05).

### 3.2 Polygenic risk score model for IHD

**Figure 2:**
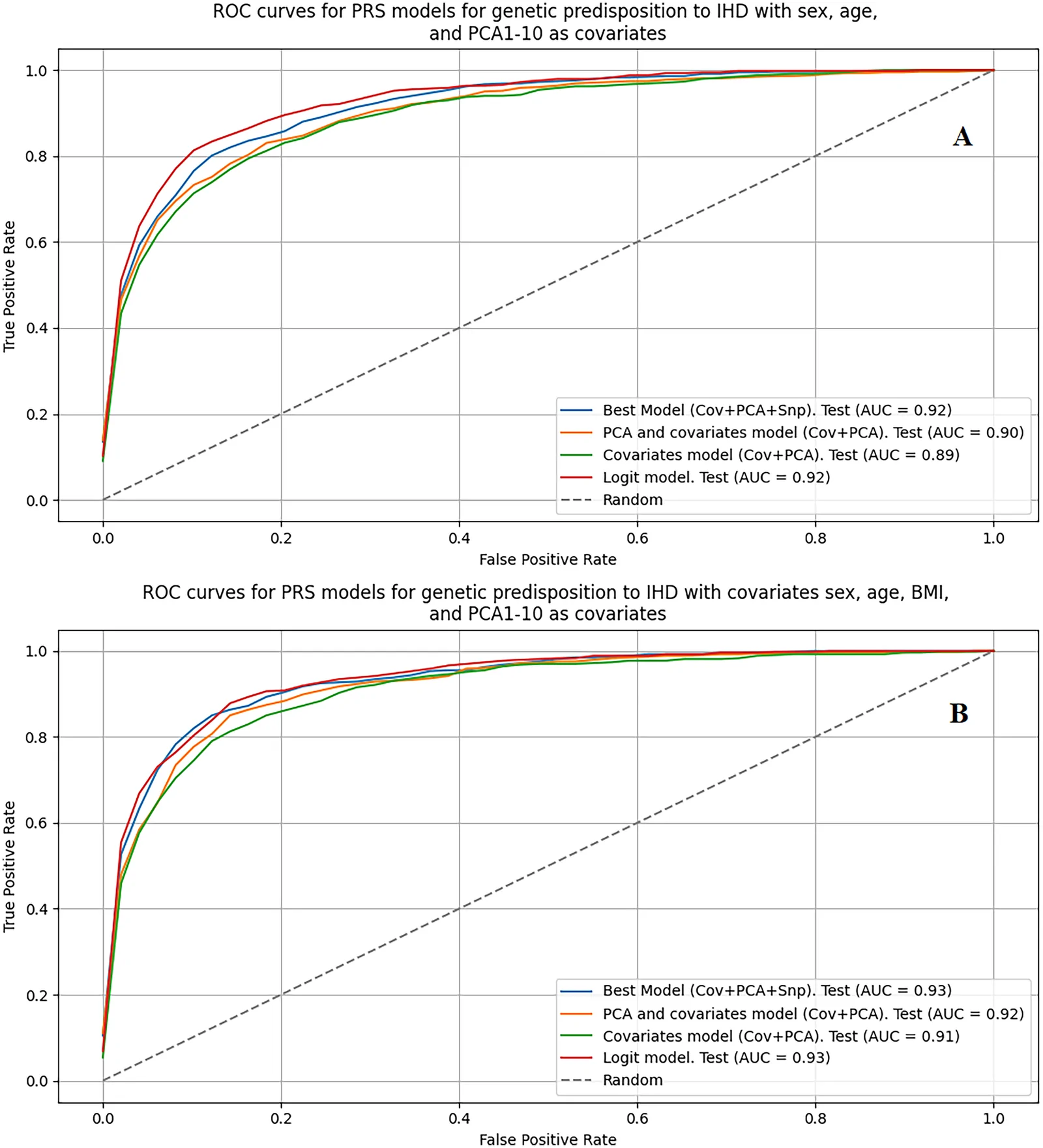
Polygenic risk score models. **(A)** ROC curves for PRS models for genetic predisposition to IHD with sex, age, and PC1-10 as covariates. **(B)** ROC curves for PRS models for genetic predisposition to IHD with covariates sex, age, BMI, and PC1-10 as covariates.

GWAS results for IHD were used in the construction of polygenic risk score models for assessing the predisposition to IHD. The best-performing PRS model based on the non-BMI-adjusted GWAS included 120 genetic variants and generated an ROC AUC of 0.92 on the test dataset (Figure 2A; Supplementary Table S3). The best-performing PRS model based on the BMI-adjusted GWAS included 80 genetic variants and generated an ROC AUC of 0.93 (Figure 2B; Supplementary Table S4).

#### 3.2.1 PRS calculations

**Figure 3:**
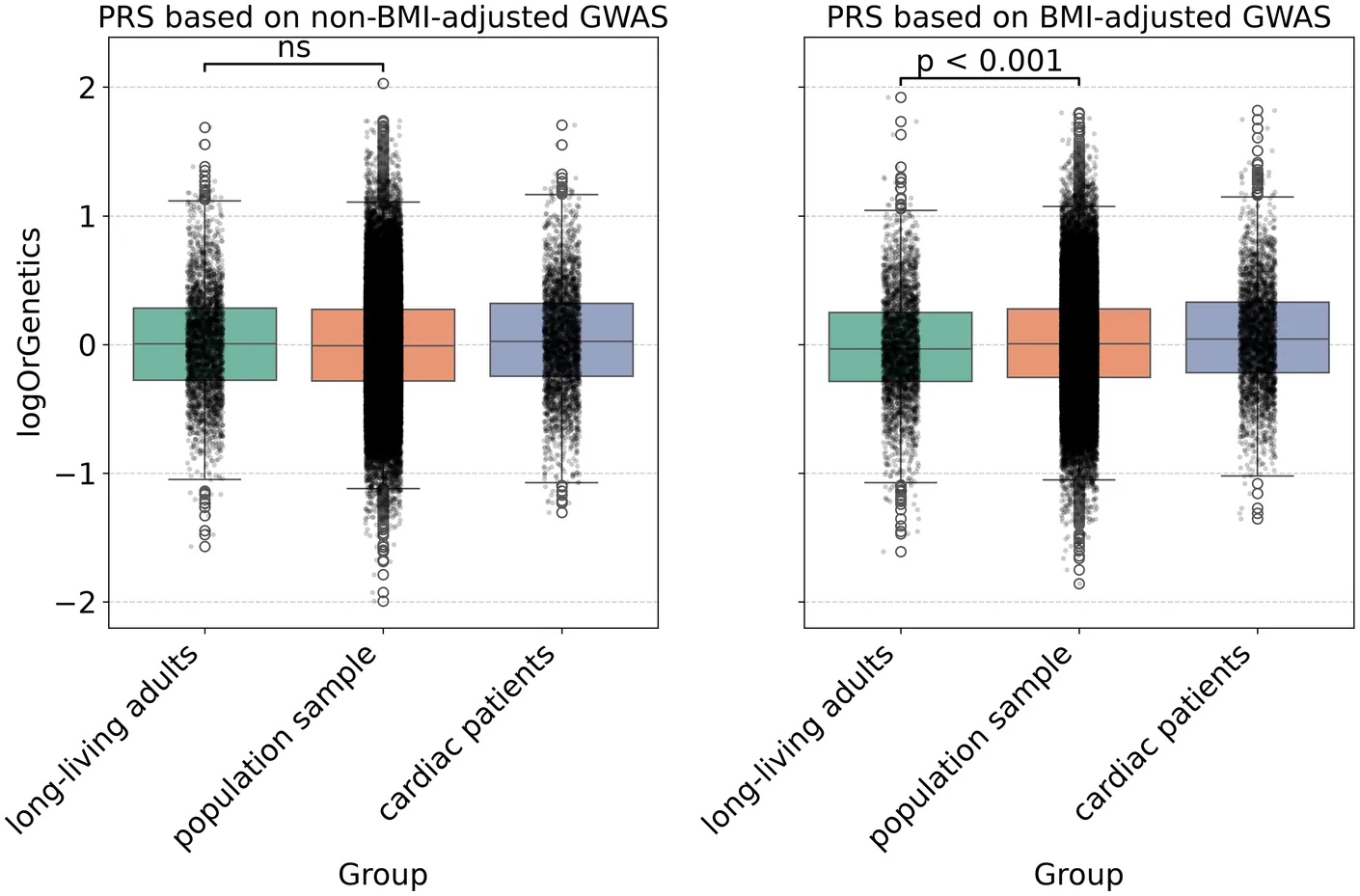
Results of PRS model testing **(A)** Distribution of genetic risk predicted by PRS based on non-BMI-adjusted GWAS. **(B)** Distribution of genetic risk predicted by PRS based on BMI-adjusted GWAS. logOrGenetics: logarithm of odds ratio for genetic risk.

PRSs for IHD were calculated for the population sample, cardiac patients, and long-living adults. Table 2 provides the clinical characteristics of the PRS cohorts. In pairwise comparison, no differences were observed in the risk of IHD between long-living adults and the population sample. The risk of IHD in cardiac patients was statistically significantly higher compared to both the population sample and long-living adults (*p* < 0.001). After adjusting for BMI, the risk of IHD in long-living adults was lower than that in the population sample (*p* < 0.001), Figure 3.

**Table 2:** Clinical characteristics of the PRS cohorts.

Characteristic	Cardiac patients (*n* = 2,996)	Population sample (*n* = 45,866)	Long-living adults (*n* = 2,870)
Age, years	63 (52–72)	55 (46–63)	92 (91–94)
Male sex, *n* (%)	1,318 (43.99%)	26,230 (57.19%)	714 (24.89%)
BMI, kg/m²	28.30 (24.98–32.14)	27.58 (24.77–30.79)	25.46 (23.11–28.34)
BMI > 30 kg/m², *n* (%)	353 (11.78%)	3,764 (8.21%)	94 (3.28%)
Hypertension, *n* (%)	1,839 (61.38%)	22,737 (49.57%)	2,586 (90.14%)
CHD, *n* (%)	840 (28.04%)	4,703 (10.25%)	541 (18.86%)
MI, *n* (%)	346 (11.55%)	1,346 (2.93%)	541 (18.86%)
Type 2 DM, *n* (%)	347 (11.58%)	3,397 (7.41%)	420 (14.64%)
Stroke, *n* (%)	300 (10.01%)	806 (1.76%)	458 (15.96%)
Total cholesterol, mmol/L	4.58 (3.77–5.47)	5.30 (4.60–6.10)	4.76 (3.92–5.57)
LDL-C, mmol/L	2.61 (1.97–3.49)	3.53 (2.82–4.27)	2.86 (2.20–3.56)
HDL-C, mmol/L	1.23 (1.03–1.47)	1.38 (1.16–1.64)	1.24 (1.03–1.51)
Triglycerides, mmol/L	1.30 (0.95–1.77)	1.39 (0.99–2.01)	1.17 (0.90–1.53)
Smoking, *n* (%)	1,103 (36.82%)	16,858 (36.75%)	323 (11.26%)

The intragroup comparison showed that the prevalence of Alzheimer's disease (*p* = 0.03) and a history of myocardial infarction (*p* = 0.04) was lower in long-living adults in the first quartile compared to those in the fourth quartile. However, after adjusting for multiple testing using the Benjamini–Hochberg procedure, these associations were no longer statistically significant.

### 3.3 Transcriptome analysis

#### 3.3.1 Transcriptome-wide association study (TWAS)

The significant genetic variants were located outside of exons, indicating their involvement in IHD pathogenesis via the regulation of gene expression rather than impaired protein structure and function. To test this hypothesis, a TWAS was conducted using polymorphism-gene expression from the GTEx Project in vascular (Artery_Aorta, Artery_Coronary, Artery_Tibial), heart (Heart_Atrial_Appendage, Heart_Left_Ventricle) and adipose tissues (Adipose_Subcutaneous, Adipose_Visceral_Omentum). Supplementary Figure S4 presents TWAS findings for all tissues. The patterns of expression changes in IHD-associated genes were similar in tissues of the same origin. Three tissue clusters were identified, each representing a specific organ or tissue type: the heart, arteries, and adipose tissue.

Only expression changes in *CDKN2B* in the aorta demonstrated a significant association with IHD after the Benjamini-Hochberg correction for multiple testing (*z*-score = 6.662; *p*-value = 2.69 × 10^−11^). Variants found significant in the GWAS and located in *CDKN2B-AS1* and nearby intergenic regions are expected to influence the expression of these gene. Supplementary Tables S5–S11 present 50 most significant genes for IHD in each tissue type.

#### 3.3.2 Differential gene expression in the clinical sample

**Figure 4:**
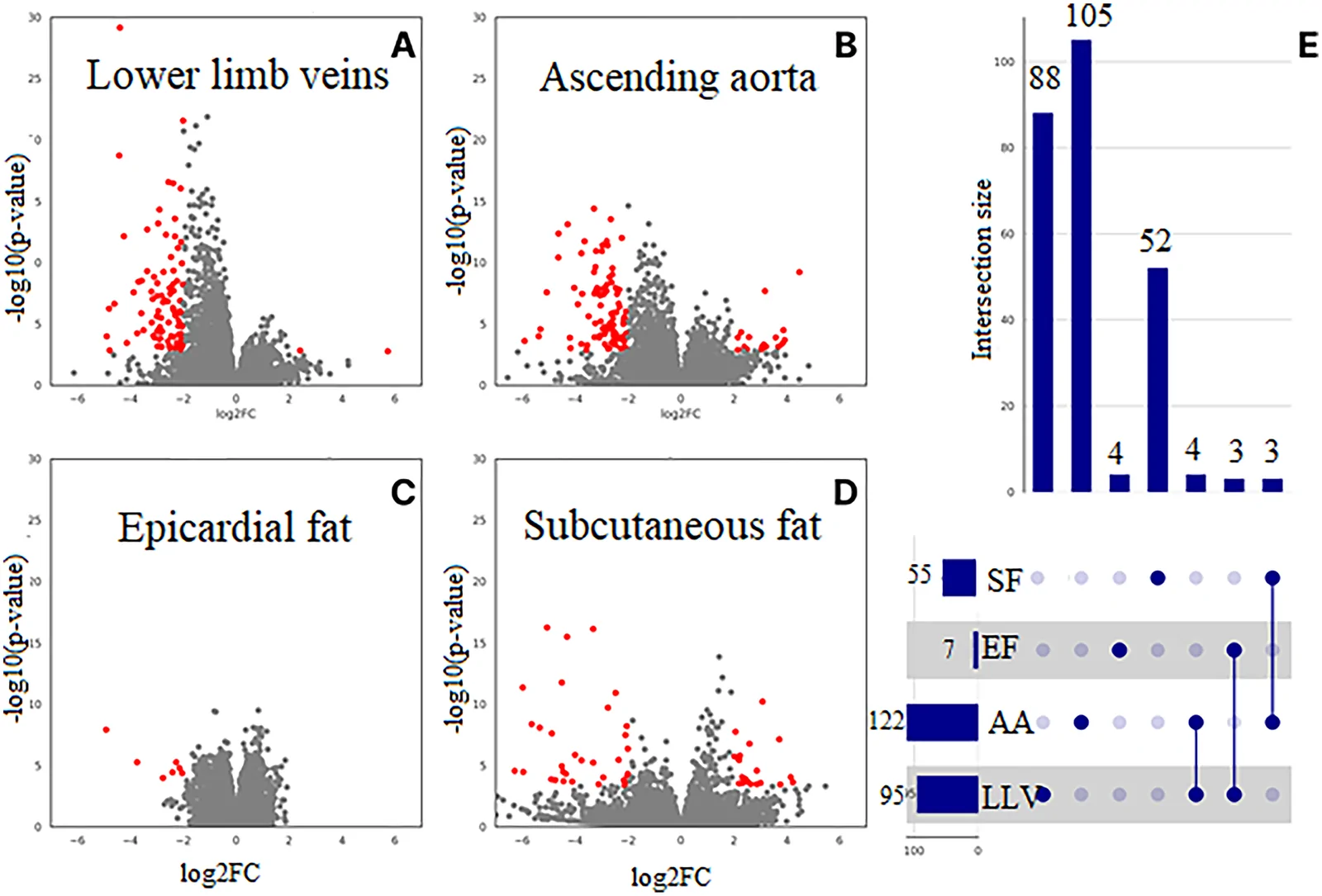
Results of differential gene expression analysis in IHD patients compared to control in four tissue types: **(A)** lower limb veins; **(B)** ascending aorta; **(C)** epicardial fat; **(D)** Subcutaneous fat. **(E)** overlapping gene sets exhibiting significant differential expression in the four tissue types.

The transcriptome analysis provided data on differential expression of genes in IHD patients and controls in four tissue types: lower limb veins (LLV), ascending aorta (AO), epicardial fat (EF), and subcutaneous fat (SCF) (Figures 4A–D).

Supplementary Table S12 presents the list of genes demonstrating significant differential expression in at least one of the four tissue types. No overlapping genes were found for all tissue types (Figure 4E). However, the *NR4A3*, *ENSG00000229644.6*, *LIF*, *and SLC7A5* genes showed significant differential expression in both LLV and AO; the *PTX3*, *MT1G*, *and MT1A* genes, in both LLV and EF; and the *PTGFR*, *SLITRK4*, *and PLA2G2A* genes, in AO and SCF.

The functional analysis yielded statistically significant results for lower limb veins and epicardial fat. The *MT1A and MT1G* genes were hypoexpressed in the epicardial fat of patients with IHD. These genes are involved in the mineral absorption metabolic pathway, which was found to be overrepresented in the ORA (padj = 0.002). The *GSTM1* gene, hyperexpressed in lower limb veins in patients with IHD, is involved in metabolic pathways, which were also overrepresented in the ORA, such as Glutathione metabolism (padj = 0.023), Metabolism of xenobiotics by cytochrome P450 (padj = 0.023), Drug metabolism (padj = 0.023), Fluid shear stress and atherosclerosis (padj = 0.023), Hepatocellular carcinoma (padj = 0.023), and Chemical carcinogenesis (padj = 0.023).

Table 3 presents more significant results obtained for genes hypoexpressed in lower limb veins.

**Table 3:** Results of the functional analysis of genes hypoexpressed in lower limb veins of IHD patients.

subcategory	ID	Description	p.adjust	Genes
Signal transduction	hsa04668	TNF signaling pathway	1.9 × 10^−5^	*SOCS3; IL6; BCL3; LIF; TNFAIP3; CCL2; CXCL2; ICAM1*
Digestive system	hsa04978	Mineral absorption	4.4 × 10^−5^	*MT2A; MT1A; MT1M; MT1G; MT1X; MT1E*
Infectious disease: parasitic	hsa05144	Malaria	0.0003	*TGFB2; IL6; CCL2; THBS1; ICAM1*
Signal transduction	hsa04630	JAK-STAT signaling pathway	0.001	*SOCS3; IL6; CDKN1A; MYC; LIF; PIM1*
Infectious disease: viral	hsa05169	Epstein–Barr virus infection	0.003	*IL6; CDKN1A; GADD45B; MYC; TNFAIP3; RELB; ICAM1*
Immune disease	hsa05323	Rheumatoid arthritis	0.003	*TGFB2; IL6; CCL2; CXCL2; ICAM1*
Infectious disease: viral	hsa05166	Human T-cell leukemia virus 1 infection	0.003	*TGFB2; IL6; ZFP36; CDKN1A; MYC; RELB; ICAM1*
Endocrine and metabolic disease	hsa04933	AGE-RAGE signaling pathway in diabetic complications	0.003	*TGFB2; IL6; PIM1; CCL2; ICAM1*
Signal transduction	hsa04064	NF-kappa B signaling pathway	0.003	*GADD45B; TNFAIP3; CXCL2; RELB; ICAM1*
Infectious disease: viral	hsa05167	Kaposi sarcoma-associated herpesvirus infection	0.007	*IL6; ZFP36; CDKN1A; MYC; CXCL2; ICAM1*
Signal transduction	hsa04068	FoxO signaling pathway	0.008	*TGFB2; IL6; CDKN1A; GADD45B*
Cancer: specific types	hsa05220	Chronic myeloid leukemia	0.008	*CDKN1A; TGFB2; GADD45B; MYC*
Signaling molecules and interaction	hsa04060	Cytokine-cytokine receptor interaction	0.008	*TGFB2; IL6; CCL8; CLCF1; LIF; CCL2; CXCL2*
Cardiovascular disease	hsa05417	Lipid and atherosclerosis	0.008	*IL6; CCL2; CXCL2; LDLR; HSPA1B; ICAM1*
Cancer: specific types	hsa05216	Thyroid cancer	0.009	*CDKN1A; GADD45B; MYC*
Cancer: specific types	hsa05210	Colorectal cancer	0.009	*CDKN1A; TGFB2; GADD45B; MYC*
Cancer: specific types	hsa05219	Bladder cancer	0.010	*CDKN1A; MYC; THBS1*
Cell growth and death	hsa04218	Cellular senescence	0.010	*TGFB2; IL6; CDKN1A; GADD45B; MYC*
Cell growth and death	hsa04110	Cell cycle	0.010	*TGFB2; CDKN1A; GADD45B; MYC; CDC7*
Immune system	hsa04657	IL-17 signaling pathway	0.010	*IL6; TNFAIP3; CCL2; CXCL2*
Infectious disease: viral	hsa05161	Hepatitis B	0.010	*TGFB2; IL6; CDKN1A; EGR3; MYC*
Signaling molecules and interaction	hsa04061	Viral protein interaction with cytokine and cytokine receptor	0.011	*IL6; CCL8; CCL2; CXCL2*
Immune system	hsa04625	C-type lectin receptor signaling pathway	0.013	*IL6; EGR3; BCL3; RELB*
Immune system	hsa04621	NOD-like receptor signaling pathway	0.017	*IL6; NAMPT; TNFAIP3; CCL2; CXCL2*
Infectious disease: bacterial	hsa05134	Legionellosis	0.017	*IL6; CXCL2; HSPA1B*
Cancer: specific types	hsa05213	Endometrial cancer	0.020	*CDKN1A; GADD45B; MYC*
Cancer: overview	hsa05202	Transcriptional misregulation in cancer	0.020	*IL6; CDKN1A; NR4A3; GADD45B; MYC*
Cancer: overview	hsa05205	Proteoglycans in cancer	0.020	*TGFB2; CDKN1A; MYC; PLAUR; THBS1*
Cancer: overview	hsa05206	MicroRNAs in cancer	0.028	*TGFB2; CDKN1A; MYC; PIM1; THBS1; EZH2*
Cell growth and death	hsa04115	p53 signaling pathway	0.033	*CDKN1A; GADD45B; THBS1*
Cancer: specific types	hsa05212	Pancreatic cancer	0.033	*CDKN1A; TGFB2; GADD45B*
Cancer: specific types	hsa05226	Gastric cancer	0.033	*TGFB2; CDKN1A; GADD45B; MYC*
Infectious disease: viral	hsa05160	Hepatitis C	0.039	*SOCS3; CDKN1A; MYC; LDLR*
Cancer: specific types	hsa05225	Hepatocellular carcinoma	0.048	*TGFB2; CDKN1A; GADD45B; MYC*
Infectious disease: viral	hsa05164	Influenza A	0.049	*SOCS3; IL6; CCL2; ICAM1*
Cancer: specific types	hsa05222	Small cell lung cancer	0.049	*CDKN1A; GADD45B; MYC*

#### 3.3.3 Comparison of predicted and observed transcriptome associations

The association between an increased expression of the *CDKN2B* gene in the ascending aorta of IHD patients, predicted by TWAS, was not confirmed by the transcriptomic analysis. The results obtained for *CDKN2B* in different tissue types were as follows:

- lower limb veins: log2FC = −1.1104, p_adj=0.113;
- ascending aorta: log2FC = 0.1296, p_adj=0.999;
- epicardial fat: log2FC = −0.3124, p_adj=0.623;
- subcutaneous fat: log2FC = 0.6476, p_adj=0.999.

Patients with IHD demonstrated higher *CDKN2B* gene expression levels in the ascending aorta compared to controls; however, this difference did not reach statistical significance. The absence of significant findings may be attributed to the insufficient sample size and the limited number of samples from patients without IHD.

## 4 Discussion

Over the past two decades, research has increasingly concentrated on the contribution of common genetic variants to the predisposition of IHD. GWASs have confirmed the polygenic nature of IHD, identifying more than 250 independent genomic loci significantly associated with this condition (20).

### 4.1 Genome-wide association study

The GWAS showed that the majority of the identified IHD-associated polymorphisms are localized within a specific cluster on chromosome 9, previously associated with this condition (21). These polymorphisms are located within the *CDKN2B-AS1* gene, the product of which is involved in epigenetic silencing of the neighboring cyclin-dependent kinase genes, *CDKN2A* and *CDKN2B*, both sited within the above cluster at 9p21.3. Reduced activity of these genes has also been linked to IHD (22). Moreover, 24 polymorphisms within this cluster are located at binding sites for up to 78 transcription factors (23). Common variants in the *CDKN2B-AS1* gene, including rs133304°C>T, have been directly linked to IHD in many populations and are considered potential biomarkers for an early IHD risk stratification. Temel et al. (24) found that the rs2977574 G and rs1333040T alleles are associated with higher serum total cholesterol and lower HDL levels in patients with IHD (*p* = 0.019 and *p* = 0.006 for rs2977574 G; *p* = 0.022 and *p* = 0.031 for rs1333040T), which is consistent with our findings.

A GWAS variation was conducted for IHD, which, alongside sex and age, included BMI as an additional covariate. Obesity is often accompanied by chronic low-grade inflammation, contributing to the progression of atherosclerosis (25). Several inflammatory pathways have been implicated in obesity, including NF-Kb, NLRP3, JNK, and TLR4. NF-*κ*B stimulates the production of adhesion molecules, such as VCAM-1 and ICAM-1, in the endothelium, thereby promoting monocyte migration (26); NLRP3 initiates an inflammatory cascade through the production of IL-1β and IL-18, aggravating inflammatory processes within atherosclerotic plaques (27); JNK increases apoptosis of endothelial and vascular smooth muscle cells, contributing to plaque instability (28); and TLR4 plays a critical role in the development of atherosclerosis by stimulating macrophages to accumulate oxidized LDL and to differentiate into foam cells, which are a primary component of atherosclerotic plaques (29).

The rs113733516 polymorphism located within an intron of the *NR5A1* gene at 9q33.3 was identified as unique to the non-BMI-adjusted GWAS. This gene encodes a key transcriptional activator involved in both steroidogenesis and gonadal differentiation (30). Polymorphisms in the *NR5A1* gene have been linked to metabolic disorders, including obesity and hyperglycemia. *In vitro* studies have demonstrated that *NR5A1* variants can interfere with the transcriptional activation of the *BDNF* gene, which produces brain-derived neurotrophic factor, a key regulator of appetite and energy balance (31). Metabolic syndrome is an important risk factor for IHD, which underlies the link between this variant and the IHD phenotype and explains its absence in the BMI-adjusted GWAS.

Six polymorphisms were identified on chromosome 6, two of which—rs140570886 and rs141766382—are located within introns of the *LPA* and *MAP3K4* genes, respectively. The *LPA* gene at loci 6q25–q26 encodes apolipoprotein(a), a component of the lipoprotein particle lipoprotein(a) [Lp(a)]. Structurally, *LPA* contains a variable number of kringle IV type 2 (KIV2) repeats, ranging from 11 to 50, which influence the molecular weight of the apo(a) protein. Elevated Lp(a) levels and the low-molecular-weight apo(a) phenotype of approximately ≤ 580 kDa are associated with an increased risk of atherosclerosis, IHD, myocardial infarction, and stroke (32). The rs10455872 and rs3798220 polymorphisms have been associated with increased plasma levels of Lp(a) and the low-molecular-weight apo(a) phenotype. Additionally, significant associations have been reported between rs3127596, rs6415084, and rs9364559 and an increased risk of IHD, while rs3127596 and rs6415084 have been linked to elevated plasma levels of Lp(a). The *MAP3K4* gene encodes mitogen-activated protein kinase 4. This protein mediates phosphorylation cascades involving various kinase proteins, influences the production of inflammatory mediators, such as IL-6, IL-1β, and COX-2, and plays a role in cell migration and epithelial-mesenchymal transition. Zhou et al. (33) reported that the progression of atherosclerosis in mice in the presence of chronic inflammation led to a 50% decrease in the expression of the divergent non-coding RNA transcribed in the antisense orientation to *MAP3K4*. Chronic vascular inflammation significantly contributes to the development of atherosclerosis. Therefore, *MAP3K4* may be implicated in the pathogenesis of IHD. Additionally, long non-coding RNA associated with *MAP3K4* (lncRNA-MAP3K4) may serve as a promising therapeutic target for IHD treatment. Furthermore, three variants—rs562851244, rs143843429, and rs186696265—found to be significant at the genome-wide level, are located near long non-coding RNA genes and have been associated with cholesterol levels, lipoprotein(a), and familial hypercholesterolemia in GWASs (34, 35). The rs55754459 polymorphism located on chromosome 6 in the 3′UTR of the *AGPAT4* gene was found significant at the genome-wide level in the BMI-adjusted GWAS for IHD. *AGPAT4* encodes a protein from the 1-acylglycerol-3-phosphate-O-acyltransferase family, which is an integral membrane protein that catalyzes the second step of *de novo* phospholipid biosynthesis by converting lysophosphatidic acid into phosphatidic acid. Temprano-Sagrera et al. (36) have reported the genome-wide significance of the rs55754459 polymorphism for the IHD.

The rs7314492 polymorphism was detected on chromosome 12 in the non-BMI-adjusted GWAS for IHD. This variant is located within the binding site for 13 transcription factors (37), approximately 80% of which function as transcriptional repressors. These include members of the Polycomb group proteins, which are key components of the “histone code”. Three of these proteins—HDAC2, RNF2, and EZH2—have been implicated in the pathogenesis of IHD, potentially through modulation of the PIP3-Akt signaling pathway via suppression of miR-22 expression. Elevated levels of miR-22 have been linked to the reduced proliferation and migration of vascular smooth muscle cells (38–40). Three of the thirteen transcription factors—EZH2, ZBTB7A, and EZH2phosphoT487—(41) are associated with the regulation of hematopoiesis and the development of the circulatory system. Another transcription factor, KLF9, is associated with lipid metabolism, while its impaired function is implicated in lipodystrophy (42). The detected polymorphism is located in the 5′UTR of the *P2RX2* gene; therefore, it can be assumed that a change in the expression level of each of the above factors may affect the regulation of its expression. The *P2RX2* gene product, the P2X2 purinoreceptor, is a cation-conducting ligand-gated ion channel. Binding of ATP to P2X2 activates the receptor, initiating processes involved in synaptic transmission between neurons and to smooth muscle cells (43). P2X2 receptors are involved in the proliferation and migration of vascular smooth muscle cells and endothelial cell regulation, as well as inflammation, making them potential therapeutic targets for IHD (44). The chr12:110905890 A>AATAT variant was detected on chromosome 12 in the GWAS for IHD with BMI as an additional covariate. This variant is an insertion in the intron region of the *CCDC63* gene (Coiled-Coil Domain Containing 63). The *CCDC63* gene encodes a protein, which is essential for spermiogenesis and sperm flagella. Near this variant, the rs10774610 (chr12:110902439T>C) and rs10849915 (chr12:110895818T>C) variants in the *CCDC63* gene were detected, which have been reported by Yamada et al. as associated with premature myocardial infarction (45).

The chr4:82798086 CG>C and rs56069116 variants were unique to the BMI-adjusted GWAS for IHD. The intronic variant chr4:82798086 CG>C is located in the *SCD5* gene, which encodes stearoyl-CoA desaturase 5 (*SCD5*). This enzyme is involved in the metabolism of saturated fatty acids and the regulation of adipose tissue distribution and plays an important role in the accumulation of visceral adipose tissue. *SCD5* also partakes in the suppression of several signaling pathways, including those involving the *AKT* gene, which has a protective effect in atherosclerosis (46, 47). Therefore, *SCD5* may be implicated in metabolic syndrome, type 2 diabetes, hypertension, and IHD (48). The rs56069116 variant is located in an intron of the *TBKBP1* (TBK1 Binding Protein 1) gene*.* The *TBKBP1* gene encodes a protein involved in the regulation of the innate immune response. It interacts with the TBK1 kinase, which plays a key role in activating the NF-*κ*B signaling pathway and producing type I interferons. Mutations in the *TBKBP1* gene may be associated with various neurodegenerative diseases, such as frontotemporal dementia, multiple sclerosis, and amyotrophic lateral sclerosis. The GWAS by Zhang et al. (49) identified associations between variants in the *TBKBP1* gene and levels of total cholesterol and LDL, suggesting that the rs56069116 variant may play a role in atherosclerosis and IHD through its impact on lipid metabolism.

This study identified five variants previously not reported as associated with IHD: rs141766382 within an intron of the *MAP3K4* gene; rs7314492 in the 5′UTR of the *P2RX2* gene; chr12:110905890 A>AATAT within an intron of the *CCDC63* gene; chr4:82798086 CG>C within an intron of the *SCD5* gene; and rs56069116 within an intron of the *TBKBP1* gene. Additionally, long non-coding RNAs, such as CDKN2B-AS1 and lncRNA-MAP3K4, and P2X2B receptors were identified as potential therapeutic targets for IHD.

### 4.2 Polygenic risk scores for IHD

Individually, single genetic variants associated with IHD have a limited effect; however, collectively, these variants can significantly contribute to the disease risk. The GWAS findings for IHD were used in developing polygenic risk scores (PRS) models predicting the predisposition to IHD. Overall, the PRS models with and without the BMI adjustment demonstrated similar performance, achieving ROC AUC values of 0.92 and 0.93, respectively. No significant differences in IHD risk were observed between the sample of long-living adults and the population sample. However, after adjusting for BMI, IHD risk was found to be statistically significantly lower in the sample of long-living adults than in the population sample. Obesity is recognized as an independent risk factor for IHD. The absence of differences in IHD risk between the two samples is likely influenced by variants associated with obesity. Furthermore, the relationship between BMI and the risk of cardiovascular disease varies across different age groups, with BMI having the greatest effect in individuals under 50 years of age, and its impact gradually decreasing in older populations. Higher BMI in older adults has been associated with lower mortality rates compared to normal BMI, a phenomenon often referred to as the “obesity paradox” (50). Observational studies have shown that a healthy lifestyle, including not smoking, maintaining a healthy weight, engaging in regular physical activity, and following a nutritious diet, is associated with a 50% relative reduction in IHD risk. Individuals with the PRSs experienced the greatest absolute risk reduction (5.6%) through adopting a healthy lifestyle (7, 51). However, current clinical practice does not routinely incorporate assessments for patients at high genetic risk of IHD, despite the potential benefits of targeted screening and preventive strategies in significantly reducing IHD risk. Integrating genetic risk factors with clinical indicators into a comprehensive risk prediction model should account for both primary and acquired risk factors. Several studies have integrated data from 10-year cardiovascular risk assessments with PRSs. However, further research is necessary to refine and adjust these integrated models to different populations (52, 53).

### 4.3 Transcriptome analysis

Considering that the majority of IHD-associated variants with genome-wide significance are located within non-coding regions of the genome, it is most probable that their primary mechanism of influence involves the regulation of gene expression. The TWAS, aimed at testing this hypothesis, showed a significant association between IHD and changes in the expression of the CDKN2B gene in the aorta after the multiple testing correction. The gene expression levels are influenced by variants located in the *CDKN2B-AS1* gene, which were found significant in the GWAS for IHD. *CDKN2B-AS1* encodes antisense RNA to the *CDKN2B* gene. Research has shown that the *CDKN2B-AS1* antisense RNA exhibits abnormal expression in a wide range of cancers. Recently, the molecular and cellular similarities between atherosclerosis and malignant neoplasms have been widely discussed, such as marked cellular plasticity, clonal expansion of certain cell subtypes (e.g., macrophages and smooth muscle cells in atherosclerosis), impaired efferocytosis, and increased proliferation due to the dysregulation of the cell cycle and resistance to apoptosis (54). Smooth muscle cells within atherosclerotic plaques acquire numerous tumor-like characteristics, including genomic instability, hyperproliferation, resistance to apoptosis, invasiveness, and activation of cancer-associated metabolic pathways (55). *CDKN2B-AS1* may be a promising therapeutic target or prognostic biomarker for both cardiovascular diseases and cancers (56).

The analysis of differential gene expression in four tissue types (lower limb veins, ascending aorta, epicardial fat, and subcutaneous fat) showed significant differences in IHD-associated gene expression patterns. No gene overlaps were identified for all four tissues. A significant decrease in the expression of the *NR4A3*, *ENSG00000229644.6*, *LIF*, and *SLC7A5* genes was observed in lower limb veins and the ascending aorta. A decrease in the expression of the *PTX3*, *MT1G*, and *MT1A* genes was observed in both lower limb veins and epicardial fat. Interestingly, the *PTGFR*, *SLITRK4*, and *PLA2G2A* exhibited a significant decrease in expression in the ascending aorta and a significant increase in subcutaneous fat. These findings could contribute to developing tissue-specific diagnostic panels; however, larger-scale studies are needed to validate these results.

An ORA was conducted to identify biological pathways associated with IHD. The *MT1A* and *MT1G* genes from the metallothionein family were hypoexpressed in IHD patients. These genes are involved in the mineral absorption pathway, which was overrepresented in the ORA. Metallothioneins are antioxidant zinc-binding proteins that have the potential to mediate cardioprotection. Disruptions in zinc homeostasis and metallothionein function may result from oxidative stress associated with cardiovascular diseases (57). The *GSTM1* gene was found to be overexpressed in lower limb veins of IHD patients. *GSTM1* encodes the antioxidant enzyme glutathione S-transferase mu-1, which is involved in the detoxification from numerous toxic compounds and endogenous products of oxidative stress. Oxidative stress plays a key role in atherogenesis, and the overexpression of this gene in IHD patients may be a compensatory mechanism (58). The analysis revealed several genes with decreased expression involved in overrepresented pathways in the lower limb veins of IHD patients. Pro-inflammatory signaling pathways (such as the TNF signaling pathway, JAK-STAT signaling pathway, NF-kappa B signaling pathway, and cytokine-cytokine receptor interaction) as well as pathways related to lipid metabolism (including lipid and atherosclerosis pathways) are known to play significant roles in the development and progression of IHD. Given the functional and structural differences between veins and arteries, the reduced expression of genes associated with these signaling pathways in veins may indicate a lesser involvement of veins in IHD-associated inflammatory processes and could also suggest the activation of local defense mechanisms (59). The differential expression analysis did not confirm the association between increased *CDKN2B* expression and IHD identified in the TWAS. *CDKN2B* exhibited higher expression levels in the ascending aorta of IHD patients compared to controls, but this difference was not statistically significant. The *CDKN2B* gene encodes a protein inhibitor of cyclin-dependent kinase 4B. This protein is involved in inhibiting the progression of the cell cycle's G1 phase. Mutations in the *CDKN2B* gene may be associated with both IHD and cancers. Notably, the *CDKN1A* gene, which belongs to the same family of genes as *CDKN2B*, was significantly hypoexpressed in lower limb veins of IHD patients. *CDKN1A* causes cell cycle arrest by inhibiting the activity of cyclin-dependent kinases. Additional mechanisms related to *CDKN1A* suggest its involvement in several processes such as apoptosis, DNA damage response, and stem cell regulation. This gene may function as either an oncogene or a tumor suppressor, with its expression levels varying across different tumor types. Considering the molecular similarities between the pathogenesis of atherosclerosis and cancers, *CDKN1A* could also be implicated in IHD. Further research may explore the possibility of repurposing cancer drugs for atherosclerotic conditions (56, 60).

## 5 Limitations

Polymorphisms in the *CDKN2B-AS1* gene are located in intronic regions, which complicates the precise assessment of their functional significance. The tissue samples analyzed were primarily collected during coronary artery bypass grafting procedures. Therefore, the transcriptome analysis may be influenced by potential sampling bias, with a focus on IHD patients in comparison to control subjects.

## 6 Conclusion

Research focusing on the genetic architecture of IHD with the adult Russian population is essential for assessing ethnicity-specific genetic predisposition to IHD and evaluating IHD risk at a population level. The obtained data indicated that the *CDKN2B-AS1* gene may be a promising candidate as a therapeutic target or prognostic biomarker for IHD. The utility of the PRS model presented in this study lies in its population specificity. However, further studies involving larger cohorts are needed to improve its accuracy. Early identification of individuals at an increased risk for IHD through genomic screening, complementing traditional risk evaluation techniques, may facilitate improved planning and implementation of preventive strategies for cardiovascular diseases.

## Data Availability

The data analyzed in this study is subject to the following licenses/restrictions: regulatory documents. Requests to access these datasets should be directed to Natalia Romanova, nsafronova@cspfmba.ru.
